# Excellent superiority and specificity of COBAS TaqMan HCV assay in an early viral kinetic change during pegylated interferon alpha-2b plus ribavirin treatment

**DOI:** 10.1186/1471-230X-10-38

**Published:** 2010-04-16

**Authors:** Eiichi Ogawa, Norihiro Furusyo, Kazuhiro Toyoda, Hiroaki Taniai, Shigeru Otaguro, Mosaburo Kainuma, Masayuki Murata, Yasunori Sawayama, Jun Hayashi

**Affiliations:** 1 Department of General Internal Medicine, Kyushu University Hospital, Fukuoka, Japan; 2 Department of Environmental Medicine and Infectious Disease, Kyushu University, Fukuoka, Japan

## Abstract

**Background:**

An early virological response (EVR) after the start of interferon (IFN) treatment for chronic hepatitis C leads to a successful virological outcome. To analyze an association between sustained virological response (SVR) and EVR by comparing TaqMan with Amplicor assays in HCV genotype 1-infected patients treated with pegylated (PEG)-IFN alpha-2b plus ribavirin (RBV).

**Methods:**

We retrospectively analyzed a total of 80 HCV genotype 1 patients (39 SVR and 41 non-SVR patients), who received an enough dosage and a complete 48-week treatment of PEG-IFN alpha-2b plus RBV. Serum HCV RNA levels were measured by both TaqMan and Amplicor assays for each patients at Weeks 2, 4, 8 and 12 after the start of the antiviral treatment.

**Results:**

Of the 80 patients with undetectable HCV RNA by Amplicor, 17 (21.3%) patients were positive for HCV RNA by TaqMan at Weeks 12. The quantification results showed that no significant difference in the decline of HCV RNA level between TaqMan and Amplicor 10-fold method assays within the initial 12 weeks of the treatment was found. However, the qualitative analysis showed significant differences of the positive predictive rates for SVR were found between TaqMan (100% at weeks 4 and 100% at weeks 8) and Amplicor (80.0% and 69.6%, respectively).

**Conclusions:**

The COBAS TaqMan HCV assay is very useful for monitoring HCV viremia during antiviral treatment to predict a SVR in HCV genotype 1 patients.

## Background

The hepatitis C virus (HCV) infection is a main cause of viral chronic hepatitis in Japan, where approximately 1.8 million patients have HCV infection. The prognosis of liver fibrosis made by wound-healing response to chronic liver injury may lead to cirrhosis [1,2].

The most current antiviral treatment for chronic HCV infection is pegylated interferon (PEG-IFN) alpha in combination with ribavirin (RBV), which has been used in worldwide [3,4]. The combination treatment has resulted in a higher rate of sustained virological response (SVR) than standard interferon (IFN) monotherapy [5,6]. The predictor of IFN treatment response such as HCV genotype, HCV RNA level, age, sex, the stage of liver fibrosis, the duration and dose of antiviral treatment and so on has been reported previously [7-9]. Otherwise, the predictive factor of antiviral efficacy after the start of antiviral treatment has been studied by HCV dynamics, which the duration of virological clearance of HCV RNA is associated with the therapeutic efficacy. Especially, an early viral response (EVR), a virological clearance by antiviral treatment within the initial 12 weeks, is significantly correlated with SVR in the treated patients [10-12].

Quantifying serum HCV RNA level helps to evaluate the efficacy and monitoring of antiviral treatment. Both qualitative and quantitative HCV RNA assay using COBAS Amplicor system (Amplicor) has been used well in clinical [13]. Recently, COBAS TaqMan HCV assay (TaqMan) equipped with highly detection sensitivity and widely measuring range has been developed, and this has enabled to measure qualitative and quantitative analysis simultaneously [14,15]. The aim of the present study is to analyze an association between SVR and EVR by comparing TaqMan with Amplicor assays in patients with HCV genotype 1 infection who has been treated with PEG-IFN alpha-2b plus RBV.

## Methods

### Patients

A retrospective study of 80 Japanese genotype 1-infected chronic hepatitis C patients who were treated with 48-week PEG-IFN alpha-2b plus RBV was done from January 2005 to July 2007, in order to analyze an association between sustained virological response (SVR) and early virological response (EVR) by comparing TaqMan with Amplicor assays. To exactly consider an association between antiviral response and viral kinetics, we selected all 80 patients who received both PEG-IFN alpha-2b 80% or over and RBV 60% or over of the target dosage of 48-week [16]. Using COBAS Amplicor HCV Monitor Test v2.0 (qualitative Amplicor), the 80 selected patients were negative for HCV RNA during the treatment and no patients with positivity HCV RNA during the treatment were included in this study.

SVR was defined as serum HCV RNA undetectable at 24 weeks after the end of treatment. Patients who had undetectable HCV RNA within the initial 12 weeks of treatment were considered to have had an EVR. Of the 80 patients, 39 achieved SVR at 24 weeks after the end of treatment, and 41 had clearance of HCV viremia during the treatment but achieved non-SVR after the end of the treatment.

All patients were satisfied the following criteria. Criteria for exclusion were: (i) clinical or biochemical evidence of hepatic decompensation, advanced cirrhosis identified by large esophageal varices (F2 or F3), history of gastrointestinal bleeding, ascites, encephalopathy, or hepatocellular carcinoma; (ii) hemoglobin level < 115 g/L, white blood cell count < 3 × 10^9^/L, and platelet count < 50 × 10^9^/L; (iii) concomitant liver disease other than hepatitis C (hepatitis B surface antigen positive or human immunodeficiency virus antibody positive); (iv) excessive active alcohol consumption (> 60 g/day converted into ethanol) or drug abuse; (v) severe psychiatric disease; and (vi) antiviral or corticosteroid therapy within 12 months prior to the enrolment. Patients who fulfilled the above criteria were recruited at the Department of General Internal Medicine, Kyushu University Hospital.

The diagnosis of chronic hepatitis and cirrhosis was based on a liver biopsy in each patient. All patients were diagnosed with chronic active hepatitis with piecemeal necrosis or fibrosis formation of portal-portal bridging. No significant differences were observed between these group patients at entry.

Clinical and biochemical characteristics of the enrolled 80 patients with chronic HCV infection are summarized in Table 1. The mean age of SVR patients was significantly lower (54.8 ± 9.3 years) than that of non-SVR patients (59.8 ± 9.7 years) (P = 0.0133). No significant differences were found in the means for body mass index, alanine aminotransferase, γ-glutamyl-transpeptidase or platelet count between SVR and non-SVR patient groups.

**Table 1:** Characteristics of 80 genotype 1-infected chronic hepatitis C patients treated with pegylated interferon alpha 2b plus ribavirin combination, classified by the treatment response

Characteristics	Total	SVR	Non-SVR	P value
	No. = 80	No. = 39	No. = 41	
Male No. (%)	36 (45.0)	17 (43.6)	19 (46.3)	0.9821
Age (years)	57.4 ± 9.8	54.8 ± 9.3	59.8 ± 9.7	0.0133
Body Mass Index (kg/m^2^)	23.0 ± 3.1	23.1 ± 3.0	23.6 ± 3.4	0.7821
Alanine aminotransferase (IU/L)	89.9 ± 65.4	93.2 ± 62.8	86.5 ± 65.7	0.7832
γ-glutamyltranspeptidase (IU/L)	59.2 ± 54.0	41.2 ± 34.2	77.3 ± 33.1	0.0548
Albumin (g/dL)	4.2 ± 0.4	4.2 ± 0.4	4.0 ± 0.4	0.7105
White blood cell count (10^9^/L)	5.6 ± 1.4	5.3 ± 1.4	5.8 ± 1.7	0.4666
Hemoglobin (g/L)	131 ± 14	133 ± 14	129 ± 14	0.2310
Platelet count (10^9^/L)	161 ± 51	167 ± 45	156 ± 55	0.1576
Creatinine (mg/dL)	0.71 ± 0.14	0.69 ± 0.16	0.70 ± 0.15	0.3376
Creatinine clearance (mL/min)	98.8 ± 27.6	100.2 ± 28.7	99.3 ± 32.3	0.4102
Histological cirrhosis No. (%)	6 (7.5)	2 (5.1)	4 (9.7)	0.6758

HCV, hepatitis C virus

Data is shown as the mean ± standard deviation.

SVR means a sustained virological response, which is defined as serum HCV RNA undetectable at 24 weeks after the end of treatment.

The study was conducted in accordance with the ethical guidelines of the Declaration of Helsinki and the International Conference on Harmonization of guidelines for good clinical practice.

### Therapeutic protocol

All patients were treated with a weight-based, 1.5 μg/kg weekly dose of subcutaneous PEG-IFN alpha-2b (PegIntron A; Schering-Plough, Osaka, Japan). In combination with PEG-IFN alpha-2b, RBV (Rebetol; Schering-Plough) was given orally at a daily dose of 600-1,000 mg based on bodyweight (600 mg for patients weighing < 60 kg, 800 mg for those weighing 60-80 kg, and 1,000 mg for those weighing > 80 kg). The length of the combined treatment was 48 weeks. The above duration and dosage are those approved by the Japanese Ministry of Health, Labor and Welfare. Patients were considered to have RBV-induced anemia if the hemoglobin level decreased to < 100 g/L. In such cases, a reduction in the dose of RBV was required. Some patients also had PEG-IFN alpha-2b induced psychological adverse effects or a decrease of white blood cell and platelet count. In such cases, a reduction in the dosage of PEG-IFN alpha-2b was required. Both PEG-IFN alpha-2b and RBV were discontinued if the hemoglobin level, white blood cell count, or platelet count fell below 85 g/dL, 1 × 10^9^/L, and 2.5 × 10^9^/L, respectively.

### Definition of SVR and EVR

SVR and EVR were defined as non-detectable HCV RNA as measured by the COBAS Amplicor HCV Monitor Test v2.0 (qualitative Amplicor) (Roche Diagnostics, Tokyo, Japan), and the results were labeled as positive or negative. The lower limit of detection was 50 IU/mL (0.5 kIU/mL: 1.7 log IU/mL) [17]. Moreover, we compared serum HCV RNA negativity by the qualitative Amplicor assay with that by TaqMan assay during the initial 12 weeks of treatment.

### Determination of HCV RNA level

During the treatment period (the initial 12 weeks: at week 2, week 4, week 8, and week 12), we retrospectively determined serum HCV RNA level by both COBAS TaqMan HCV assay (TaqMan) (Roche Diagnostics) and COBAS Amplicor HCV Monitor Test v2.0 using the 10-fold dilution method (Amplicor 10-fold method) (Roche Diagnostics) in each patient. The TaqMan has a lower limit of quantitation of 15 IU/mL and an outer limit of quantitation of 6.9 × 10^7 ^IU/mL (1.2 to 7.8 log IU/mL referred to log_10 _units/mL) [14,15]. Therefore, TaqMan assay is able to do both qualitative and quantitative analysis for HCV RNA. The Amplicor 10-fold method has a lower limit of quantitation of 5,000 IU (5 kIU/mL) and an outer limit of quantitation of 5,100,000 IU (5,100 kIU/mL) [18]. To compare serum HCV RNA level by TaqMan assay with Amplicor 10-fold method assay, we transformed the level by Amplicor 10-fold method assay (kIU/mL) into the logarithmic level (log IU/mL). Therefore, the range of Amplicor 10-fold method is 3.7 to 6.7 log IU/mL.

### Determination of HCV genotype

HCV genotype was determined using type-specific primers from the core region of the HCV genome. The protocol for genotyping was carried out as described earlier [19].

### Statistical analysis

Statistical analysis was done with BMDP statistical software for the IBM 3090 system computer (BMBD Statistical Software, Inc., Los Angeles, CA, USA) for the IBM (Yorktown Heights, NY) 3090 computer system. Continuous data were expressed as mean values, mean ± standard deviation (SD), or values ± standard error (SE) of the mean. The paired t-test, unpaired t-test, Mann-Whitney U test or Kruskal-Wallis non-parametric analysis of variance was used to compare HCV dynamics. A P value less than 0.05 was regarded as statistically significant.

## Results

### The correlation of pretreatment HCV RNA levels between TaqMan and Amplicor 10-fold method assays

The relationship of pretreatment HCV RNA levels between TaqMan and Amplicor 10-fold method assays was studied in 39 SVR and 41 non-SVR patients infected with genotype 1. The levels by TaqMan ranged from 4.4 to 7.2 log IU/mL (median 6.1 log IU/mL) and those by Amplicor 10-fold method ranged from 5.0 to 6.7 log IU/mL (median 6.0 log IU/mL). We found a significantly positive correlation in the pretreatment HCV RNA level between TaqMan and Amplicor 10-fold method assays (r = 0.849, P < 0.0001).

**Figure 1:**
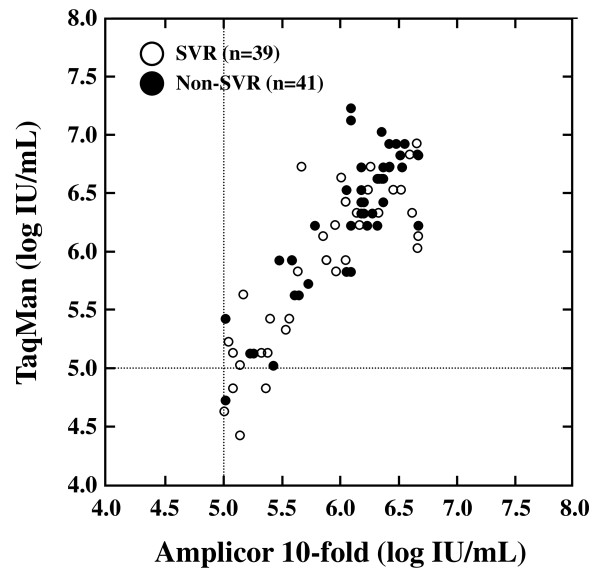
**Correlation of pretreatment hepatitis C virus (HCV) RNA levels between TaqMan and Amplicor 10-fold method assays, classified by sustained virological response (SVR) and non-SVR**. Open and closed circles mean SVR and non-SVR patients, respectively.

Figure 1 shows pretreatment levels of HCV RNA classified by viral response (SVR or non-SVR). In TaqMan assay, the 39 SVR patients had significantly lower pretreatment HCV RNA level (median 5.89 log IU/mL) than the 41 non-SVR patients (median 6.25 log IU/mL) (P = 0.0191). However, Amplicor 10-fold method assay showed no significant difference of pretreatment HCV RNA level between the SVR (median 5.91 log IU/mL) and non-SVR (median 6.09 log IU/mL) patients (P = 0.0929). Therefore, pretreatment HCV RNA by TaqMan assay may be a predictive factor for SVR, but not by Amplicor 10-fold method assay among patients with HCV genotype 1.

### The comparison with TaqMan and Amplicor 10-fold method assays in HCV dynamics within the initial 12 weeks of PEG-IFN plus RBV combined treatment

**Figure 2:**
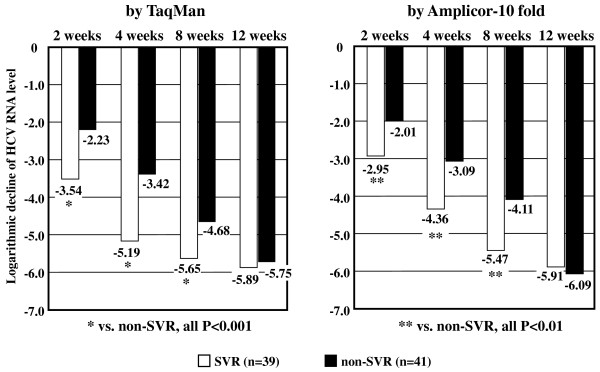
**The logarithmic declines from pretreatment hepatitis C virus (HCV) RNA by TaqMan and Amplicor 10-fold method assays within the initial 12 weeks during pegylated interferon alpha plus ribavirin treatment**. White and black bars mean sustained virological response (SVR) and non-SVR patients, respectively.

The relationship of HCV dynamics within the initial 12 weeks of the combined treatment between TaqMan and Amplicor 10-fold method assays was studied in 39 SVR and 41 non-SVR patients. Figure 2 shows the difference in the logarithmic decline from pretreatment HCV RNA level within the initial 12 weeks of the treatment between SVR and non-SVR patients by TaqMan and Amplicor 10-fold method assays. The logarithmic declines of HCV RNA levels by TaqMan in SVR patients (-3.54, -5.19, and -5.65 at weeks 2, 4, and 8, respectively) were significantly higher than those in non-SVR patients (-2.23, -3.42, and -4.68 at weeks 2, 4, and 8, respectively) (P < 0.0001, P < 0.001, and P = 0.0012, respectively), except for at weeks 12 (-5.89 and -5.75, P = 0.3936). Similarly, the logarithmic declines of HCV RNA levels by Amplicor 10-fold method assay in SVR patients (-2.95, -4.36, and -5.47 at weeks 2, 4, and 8, respectively) were significantly higher than non-SVR patients (-2.01, -3.09, and -4.11 at weeks 2, 4, and 8, respectively) (P = 0.00281, P = 0.00021, and P = 0.0006, respectively), except for at weeks 12 (-5.91 and -6.09, P = 0.0929). No significant difference in monitoring the HCV dynamics during the initial 12 weeks administration of the combined treatment was found between TaqMan and Amplicor 10-fold method assays.

### The comparison with TaqMan and qualitative Amplicor assays in HCV RNA negativity within the initial 12 weeks of PEG-IFN plus RBV combined treatment

The undetectable HCV RNA by TaqMan was found in all of 39 SVR patients (100%) but only in 24 of 41 non-SVR patients (58.5%), while that by qualitative Amplicor was found in all of 39 SVR and 41 non-SVR patients. TaqMan reduced the numbers of undetectable HCV RNA in the group of non-SVR.

**Figure 3:**
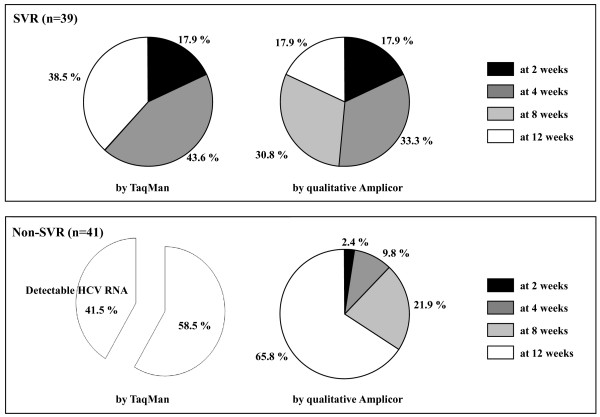
**The distribution of timing of HCV RNA undetectable after the initiation of treatment at weeks 2, 4, 8, and 12 in comparison with TaqMan and qualitative Amplicor assays**. SVR, sustained virological response.

Figure 3 shows the distribution of timing of HCV RNA undetectable after the initiation of treatment at weeks 2, 4, 8, and 12 in comparison with TaqMan and qualitative Amplicor assays. Among 39 SVR patients, the distributions of timing of detectable HCV RNA were 17.9% (7/39), 43.6% (17/39), 0% (0/39), and 38.5% (15/39) by TaqMan and 17.9% (7/39), 33.3% (13/39), 30.8% (12/39), and 17.9% (7/39) by qualitative Amplicor assay at 2, 4, 8, and 12 weeks, respectively. There was no significant difference in timing of undetectable HCV RNA between TaqMan and qualitative Amplicor assays in the group of SVR. Among 41 non-SVR patients, the distribution of timing of detectable HCV RNA by TaqMan was 58.5% (24/41) at 12 weeks, and those by qualitative Amplicor assay were 2.4% (7/41), 9.8% (4/41), 21.9% (9/41), and 65.8% (27/41) at 2, 4, 8, and 12 weeks, respectively. Patients of non-SVR had apparent delayed timing of undetectable HCV RNA, and the tendency was more frequently found by TaqMan than by qualitative Amplicor assay.

### The comparison with TaqMan and qualitative Amplicor assays for positive predictive value (PPV) rates of SVR in patients with EVR

**Figure 4:**
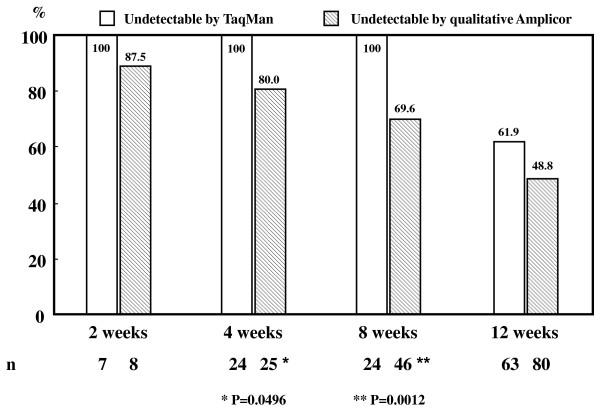
**Positive predictive value rates for sustained virological response within the initial 12 weeks during pegylated interferon alpha plus ribavirin treatment, classified by TaqMan and qualitative Amplicor assays**.

The PPV rates for SVR were calculated within the initial 12 weeks during the combined treatment, based on HCV RNA negativity by TaqMan and qualitative Amplicor assays, respectively. Figure 4 shows significant differences of the initial 12-week PPV rates for SVR were found between TaqMan assay (at weeks 4 and 8, 100% and 100%, respectively) and qualitative Amplicor (80.0% and 69.6%, respectively) assays. To predict SVR when patients have clearance of viremia, TaqMan assay is more useful than qualitative Amplicor assay in analysis of the early stage (within 8 weeks) of HCV dynamics.

## Discussion

Measurement of serum HCV RNA level is absolutely imperative for the treatment of chronic HCV infection, because this is an effective predictive and indicative factor for the response of IFN therapy and duration of therapy [7,9,10,20]. A more useful analysis is the time to first undetectability and its relation to the end of treatment response, relapse rate and SVR [12]. Therefore, an advanced technique for detecting serum HCV RNA during the antiviral treatment needs a high degree of accuracy. The ideal molecular analysis for HCV RNA level has to be sensitive, accurate and have a broad dynamic range to monitor viral load changes during antiviral therapy. TaqMan assay is useful for simultaneously analyzing qualitative and quantitative HCV RNA level in serum [14,15]. The present study included only patients infected with chronic HCV genotype 1, who received a full of 48-week treatment of PEG-IFN plus RBV combination with target dosages of both drugs (PEG-IFN alpha-2b 80% or over and RBV 60% or over of the above prescribed dosage). We believed that analyzing the exact response factors for antiviral treatment should be considered only under this enough dosage of the combined treatment [16]. The present study showed the superiority of TaqMan assay over Amplicor assay for analyzing the early prediction of virologic response to antiviral therapy.

TaqMan assay, the performance of a fully automated system based on the real-time PCR technology, was evaluated for nucleic acid extraction from plasma. This results in enhanced user convenience, a great reduction in labor requirements minimizing hands-on time and a decrease the risk of sample contaminations. TaqMan assay was extremely sensitive with a linear dynamic range up to 6.6 log IU/mL (0.015-69,000 kIU/mL: 1.2-7.8 log IU/mL) [14,15]. Otherwise, Amplicor 10-fold method assay had a narrower linear dynamic range up to 3.0 log IU/mL (5-5,000 kIU/mL: 3.7-6.7 log IU/mL) than TaqMan assay and qualitative Amplicor assay, the lower limit of detection was 50 IU/mL (1.7 log IU/mL) [17]. These different ranges can explain significant differences in the pretreatment HCV RNA levels and the reduction rates of HCV RNA levels during an antiviral treatment between TaqMan and Amplicor assays.

Previous studies demonstrated that PEG-IFN plus RBV treatment dramatically increased SVR rate in patients with HCV infection and thus are currently the gold standard of treatment [3,4,7]. The most significant predictors of SVR to IFN treatment for patients with chronic HCV infection are absence of severe fibrosis or cirrhosis, non-genotype 1 and pretreatment serum low HCV RNA level [7,21,22]. With regard to virological factor after the start of antiviral treatment, early clearance of HCV RNA, or rapid decline of HCV RNA level during the early treatment duration is predictive of SVR among patients treated with IFN treatment [10,12,20]. For monitoring of the antiviral response to IFN treatment, both reverse-transcription PCR and branched DNA have been developed and have become available for clinical use [18,23,24]. These assays are especially useful because the early monitoring of favorable viral kinetics has a direct bearing on the possibility of a sustained response by IFN treatment. Actually, our findings showed that pretreatment HCV RNA by TaqMan assay may be a predictive factor for SVR, but not by Amplicor 10-fold method assay, and that TaqMan assay is more useful than qualitative Amplicor assay in analysis of the early stage (at the initial 4 and 8 weeks) of HCV dynamics, which is most related with SVR. Early prediction of early virologic response to IFN based treatment can help identify patients who are unlikely to have SVR and allow clinicians to discontinuation of treatment, saving patients the drug-induced adverse events and cost of additional treatment.

Our results suggested that the reduction rates of HCV RNA levels were significantly higher than those in non-SVR patients both in TaqMan and Amplicor 10-fold method assays for patients having achieved SVR. However, in our analysis, significant differences of the initial 4 and 8 week PPV for SVR were found between Amplicor and TaqMan assays. PPV for SVR at 4 and 8 week was 100% and 100% respectively in TaqMan assay, otherwise PPV for SVR was 80.0% and 69.6% respectively in qualitative Amplicor assay. In addition to EVR, within the initial 12 weeks undetectability of HCV RNA of the antiviral treatment, the critical role of rapid virological response (RVR), within the initial 4 weeks undetectability, on the SVR patients with genotype 1 and non-1 infection has been recently emphasized [7,9,16]. In fact, our findings showed more advantage using TaqMan assay on the PPV for SVR, especially at weeks 4 and 8, than Amplicor assay. Apparently, TaqMan assay is more accurate than Amplicor assay in analysis of the early stage (within 8 weeks) of HCV dynamics. PEG-IFN plus RBV treatment contributed to improve rates of SVR, but this rate is still lower for patients with HCV genotype 1 infection. Especially, most of Japanese patients with chronic hepatitis C who are candidate for antiviral treatment are relatively older than other countries. As a result, a relapse rate with EVR was a little high regardless of adequate treatment. Therefore, analysis of HCV dynamics after the antiviral treatment, especially for these patients, is very important.

The present study showed that 21.3% of the patients with undetectable HCV RNA by qualitative Amplicor assay were positive for TaqMan assay within the initial 12 weeks during PEG-IFN plus RBV treatment. It is possible that qualitative Amplicor assay reveals late responders by the combined treatment in comparison with TaqMan assay. Berg and colleagues suggested extended treatment duration was recommended for patients with SVR as HCV RNA positive at week 12 but negative at week 24 [25]. About such patients, we need to extend the duration of the combination treatment of PEG-IFN alpha-2b plus RBV from 48 weeks to 72 weeks by an appearance of TaqMan assay.

## Conclusions

In conclusion, the COBAS TaqMan HCV assay is useful for monitoring HCV viremia during antiviral treatment to predict a SVR in patients with chronically infected HCV genotype 1.

## Competing interests

The authors declare that they have no competing interests.

## Authors' contributions

EO performed the literature review, collected the clinical data and drafted the manuscript. NF, KT, HT, SO, MK, MM and YS collected the clinical data. NF participated in the design of the study and performed the statistical analysis. NF and JH revised the manuscript. All authors read and approved the final manuscript.

## Pre-publication history

The pre-publication history for this paper can be accessed here:

http://www.biomedcentral.com/1471-230X/10/38/prepub

## References

[B1] Hayashi J Kishihara Y Yamaji K Furusyo N Yamamoto T Pae Y Etoh Y Ikematsu H Kashiwagi S Hepatitis C viral quasispecies and liver damage in patients with chronic hepatitis C virus infection Hepatology 1997 25 697 701 10.1002/hep.510250334 9049221

[B2] Benvegnu L Gios M Boccato S Alberti A Natural history of compensated viral cirrhosis: a prospective study on the incidence and hierarchy of major complications Gut 2004 53 744 9 10.1136/gut.2003.020263 15082595 PMC1774055

[B3] Lindsay KL Trepo C Heintges T Shiffman ML Gordon SC Hoefs JC Schiff ER Goodman ZD Laughlin M Yao R Albrecht JK Hepatitis Interventional Therapy Group A randomized, double-blind trial comparing pegylated interferon alfa-2b to interferon alfa-2b as initial treatment for chronic hepatitis C Hepatology 2001 34 395 403 10.1053/jhep.2001.26371 11481625

[B4] Chander G Sulkowski MS Jenckes MW Torbenson MS Herlong HF Bass EB Gebo KA Treatment of chronic hepatitis C: a systematic review Hepatology 2002 36 S135 44 10.1002/hep.1840360718 12407587

[B5] McHutchison JG Gordon SC Schiff ER Shiffman ML Lee WM Rustgi VK Goodman ZD Ling MH Cort S Albrecht JK Interferon alfa-2b alone or in combination with ribavirin as initial treatment for chronic hepatitis C. Hepatitis Interventional Therapy Group N Engl J Med 1998 339 1485 92 10.1056/NEJM199811193392101 9819446

[B6] Poynard T Marcellin P Lee SS Niederau C Minuk GS Ideo G Bain V Heathcote J Zeuzem S Trepo C Albrecht J Randomised trial of interferon alpha2b plus ribavirin for 48 weeks or for 24 weeks versus interferon alpha2b plus placebo for 48 weeks for treatment of chronic infection with hepatitis C virus. International Hepatitis Interventional Therapy Group (IHIT) Lancet 1998 352 1426 32 10.1016/S0140-6736(98)07124-4 9807989

[B7] Furusyo N Murata M Hayashi J Jirillo E Interferon treatment of hepatitis C virus infection: From basic biology to clinical application Hepatitis C virus disease: Immunology and Clinical Applications 2008 New York: Springer Science + Business Media, LLC 148 67 full_text

[B8] Hayashi J Kishihara Y Ueno K Yamaji K Kawakami Y Furusyo N Sawayama Y Kashiwagi S Age-related response to interferon alfa treatment in women vs men with chronic hepatitis C virus infection Arch Intern Med 1998 158 177 81 10.1001/archinte.158.2.177 9448556

[B9] Furusyo N Katoh M Tanabe Y Kajiwara E Maruyama T Shimono J Sakai H Nakamuta M Nomura H Matsumoto A Shimoda S Takahashi K Azuma K Hayashi J Kyusyu University Liver Disease Study Group Interferon alpha plus ribavirin combination treatment of Japanese chronic hepatitis C patients with HCV genotype 2: a project of the Kyushu University Liver Disease Study Group World J Gastoroenterol 2006 12 784 90 10.3748/wjg.v12.i5.784 PMC4066133 16521196

[B10] Davis GL Wong JB McHutchison JG Manns MP Harvey J Albrecht J Early virologic response to treatment with peginterferon alfa-2b plus ribavirin in patients with chronic hepatitis C Hepatology 2003 38 645 52 10.1053/jhep.2003.50364 12939591

[B11] Lee SS Heathcote EJ Reddy KR Zeuzem S Fried MW Wright TL Pockros PJ Häussinger D Smith CI Lin A Pappas SC Prognostic factors and early predictability of sustained viral response with peginterferon alfa-2a (40KD) J Hepatol 2002 37 500 6 10.1016/S0168-8278(02)00211-8 12217604

[B12] Ferenci P Fried MW Shiffman ML Smith CI Marinos G Gonçales FL Jr Häussinger D Diago M Carosi G Dhumeaux D Craxì A Chaneac M Reddy KR Predicting sustained virological responses in chronic hepatitis C patients treated with peginterferon alfa-2a (40 KD)/ribavirin J Hepatol 2005 43 425 33 10.1016/j.jhep.2005.04.009 15990196

[B13] Gerken G Rothaar T Rumi MG Soffredini R Trippler M Blunk MJ Butcher A Soviero S Colucci G Performance of the COBAS AMPLICOR HCV MONITOR test, version 2.0, an automated reverse transcription-PCR quantitative system for hepatitis C virus load determination J Clin Microbiol 2000 38 2210 4 10834978 10.1128/jcm.38.6.2210-2214.2000 PMC86766

[B14] Sandres-Sauné K Abravanel F Nicot F Peron JM Alric L Boineau J Pasquier C Izopet J Detection and quantitation of HCV RNA using real-time PCR after automated sample processing J Med Virol 2007 79 1821 6 10.1002/jmv.21003 17935166

[B15] Sizmann D Boeck C Boelter J Fischer D Miethke M Nicolaus S Zadak M Babiel R Fully automated quantification of hepatitis C virus (HCV) RNA in human plasma and human serum by the COBAS AmpliPrep/COBAS TaqMan system J Clin Virol 2007 38 326 33 10.1016/j.jcv.2006.12.021 17344093

[B16] Furusyo N Kajiwara E Takahashi K Momura H Tanabe Y Masumoto A Maruyama T Nakamuta M Enjoji M Azuma K Shimono J Sakai H Shimoda S Hayashi J Kyushu University Liver Disease Study (KULDS) Group Association between the treatment length and cumulative dose of pegylated interferon alpha-2b plus ribavirin and their effectiveness as a combination treatment for Japanese chronic hepatitis C patients: Project of the Kyushu University Liver Disease Study Group J Gastroenterol Hepatol 2008 23 1094 104 10.1111/j.1440-1746.2008.05319.x 18248381

[B17] Lee SC Antony A Lee N Leibow J Yang JQ Soviero S Gutekunst K Rosenstraus M Improved version 2.0 qualitative and quantitative AMPLICOR reverse transcription-PCR tests for hepatitis C virus RNA: calibration to international units, enhanced genotype reactivity, and performance characteristics J Clin Microbiol 2000 38 4171 9 11060086 10.1128/jcm.38.11.4171-4179.2000 PMC87559

[B18] Ogawa E Furusyo N Toyoda K Takeoka H Otaguro S Hamada M Murata M Sawayama Y Hayashi J Transient elastography for patients with chronic hepatitis B and C virus infection: Non-invasive, quantitative assessment of liver fibrosis Hepatol Res 2007 37 1002 10 10.1111/j.1872-034X.2007.00160.x 17608672

[B19] Simmonds P Holmes SC Cha TA Chan SW McOmish S Irvine B Beall E Yap PL Kolberg J Urdea MS Classification of hepatitis C virus into six major genotypes and a series of subtypes by phylogenetic analysis of the NS-5 region J Gen Virol 1993 74 2391 9 10.1099/0022-1317-74-11-2391 8245854

[B20] Neumann AU Lam NP Dahari H Gretch DR Wiley TE Layden TJ Perelson AS Hepatitis C viral dynamics in vivo and the antiviral efficacy of interferon-alpha therapy Science 1998 282 103 7 10.1126/science.282.5386.103 9756471

[B21] Strader DB Wright T Thomas DL Seeff LB American Association for the Study of Liver Disease. Diagnosis, management, and treatment of hepatitis C Hepatology 2004 39 1147 71 10.1002/hep.20119 15057920

[B22] Alberti A Benvegnu L Management of hepatitis C J Hepatol 2003 36 S104 18 10.1016/S0168-8278(03)00008-4 12591189

[B23] Hayashi J Kawakami Y Nabeshima A Kishihara Y Furusyo N Sawayama Y Kinukawa N Kashiwagi S Comparison of HCV RNA levels by branched DNA probe assay and by competitive polymerase chain reaction to predict effectiveness of interferon treatment for patients with chronic hepatitis C virus Dig Dis Sci 1998 43 384 91 10.1023/A:1018874910195 9512135

[B24] Furusyo N Hayashi J Kashiwagi K Nakashima H Nabeshima S Sawayama Y Kinukawa N Kashiwagi S Hepatitis C virus (HCV) RNA level determined by second-generation branched-DNA probe assay as predictor of response to interferon treatment in patients with chronic HCV viremia Dig Dis Sci 2002 47 535 42 10.1023/A:1017955700585 11911338

[B25] Berg T von Wagner M Nasser S Sarrazin C Heintges T Gerlach T Buggisch P Goeser T Rasenack J Pape GR Schmidt WE Kallinowski B Klinker H Spengler U Martus P Alshuth U Zeuzem S Extended treatment duration for hepatitis C virus type 1: comparing 48 versus 72 weeks of peginterferon-alfa-2a plus ribavirin Gastroenterology 2006 130 1086 97 10.1053/j.gastro.2006.02.015 16618403

